# Spatial Chromosome Organization and Adaptation of *Escherichia coli* under Heat Stress

**DOI:** 10.3390/microorganisms12061229

**Published:** 2024-06-19

**Authors:** Xu-Ting Wang, Bin-Guang Ma

**Affiliations:** Hubei Key Laboratory of Agricultural Bioinformatics, College of Informatics, Huazhong Agricultural University, Wuhan 430070, China; wxtmonica@webmail.hzau.edu.cn

**Keywords:** *Escherichia coli*, 3D genome, transcriptome, thermal adaptation, chromosome conformation

## Abstract

The spatial organization of bacterial chromosomes is crucial for cellular functions. It remains unclear how bacterial chromosomes adapt to high-temperature stress. This study delves into the 3D genome architecture and transcriptomic responses of *Escherichia coli* under heat-stress conditions to unravel the intricate interplay between the chromosome structure and environmental cues. By examining the role of macrodomains, chromosome interaction domains (CIDs), and nucleoid-associated proteins (NAPs), this work unveils the dynamic changes in chromosome conformation and gene expression patterns induced by high-temperature stress. It was observed that, under heat stress, the short-range interaction frequency of the chromosomes decreased, while the long-range interaction frequency of the Ter macrodomain increased. Furthermore, two metrics, namely, Global Compactness (GC) and Local Compactness (LC), were devised to measure and compare the compactness of the chromosomes based on their 3D structure models. The findings in this work shed light on the molecular mechanisms underlying thermal adaptation and chromosomal organization in bacterial cells, offering valuable insights into the complex inter-relationships between environmental stimuli and genomic responses.

## 1. Introduction

In cellular organisms, chromosomes undergo significant compression to fit within the confines of the cell. The mechanisms by which organisms achieve this compression while still enabling vital processes like replication and transcription remain elusive. The development of chromosome conformation capture (3C) technologies has provided crucial support for analyzing chromosome organization in vivo [1]. Unlike eukaryotes, bacteria lack a nucleus encased in a nuclear membrane, resulting in DNA organization into nucleoids. Recently, there has been a growing application of 3C technologies to investigate the structural organization of bacterial nucleoids [2,3,4]. Macrodomain organization is a prevalent feature across bacterial nucleoids [5,6,7]. The Ori and Ter macrodomains in *E. coli* were initially identified using fluorescence in situ hybridization (FISH) [5], and their uneven distribution within the circular chromosome was directly visualized through fluorescence microscopy [8]. Global 3C data have validated the presence of four macrodomains and two non-structural regions in logarithmically growing cells [9,10]. Macrodomain formation is believed to be closely linked to Structural Maintenance of Chromosome (SMC) proteins. Homologs of SMC proteins in *E. coli* include MukBEF proteins, which are implicated in chromosome condensation over long distances, extending beyond the Ter macrodomain [11].

The 3C coupled with sequencing (3C-seq) data analysis has revealed the presence of 31 chromosome interaction domains (CIDs) in *E. coli* [9]. It is hypothesized that the macrodomains of *E. coli* may be comprised of nested CIDs, with a significantly higher frequency of interactions occurring within the CIDs compared to the interactions between them [12]. Some studies suggest that CID formation in *E. coli* may be stochastic and subject to variation under different growth conditions [13], with the underlying mechanism remaining elusive. It is speculated that CID formation may be influenced by factors such as DNA supercoiling, macromolecular crowding, and transcription processes. Nucleoid-associated proteins (NAPs) play a pivotal role in organizing the nucleoid structure of *E. coli*, with 12 common NAPs identified, including Fis, HU, H-NS, INF, and StpA [14]. Super-resolution fluorescence microscopy reveals the following distinct localization patterns: H-NS exhibits a clustered distribution, while HU, Fis, IHF, and StpA are dispersed throughout the nucleoid [15]. The 3C data indicate that H-NS predominantly regulates short-distance interactions, whereas HU and Fis modulate long-distance interactions through distinct mechanisms [9]. Additionally, the interactions between NAPs and DNA, alongside the concentration of metabolites, proteins, water, and salt in the cytoplasm, influence the solvent properties of the cytoplasm, thereby impacting the nucleoid spatial organization [16,17,18,19,20,21].

The heat-stress response in *E. coli* has garnered significant attention since its inception [22,23,24,25,26]. Traditional research methodologies have elucidated numerous molecular mechanisms underlying heat-response regulation [26,27,28,29], and these findings have found broad application, particularly in synthetic biology [25,30]. A previous investigation has identified a correlation between the expression levels of heat stress-responsive genes and their sequence properties [28]. The supercoil structure of bacterial DNA is influenced by transcription levels [31], and changes in temperature affect transcription levels and temperature-sensitive mRNA conformation [26]. However, it remains unclear how temperature alterations induce changes in the chromosome structure, potentially affecting the expression levels of NAPs, and how the variations in the NAP expression levels reciprocally influence chromosome conformation in *E coli*. To address these questions, we need to study the 3D genome under heat-stress conditions.

In this study, we employed 3C-seq technology to elucidate the chromosome conformations of *E. coli* under normal-temperature (37 °C) and high-temperature (45 °C) conditions. Additionally, RNA-seq technology was adopted to assess the transcription levels of *E. coli* genes across varying temperatures. Through the integration of 3D genomic and transcriptomic analyses, we observed significant alterations in both the transcription levels and the 3D organization of the *E. coli* nucleoid in response to high-temperature stress. Specifically, we observed a decrease in the interaction frequency between DNA segments within a range of approximately 200 kb, accompanied by a relative increase in the interaction frequency among DNA segments spanning over 1000 kb in the high-temperature environment. Furthermore, based on 3D structural models, the analysis of Global Compactness (GC) revealed a trend toward increased chromosome compaction under high-temperature stress, while Local Compactness (LC) exhibited distinct changes across different chromosome regions. Through the comprehensive analysis of gene transcription levels and chromosome structural features, we uncovered a close association between alterations in chromosome organization and changes in the transcription level induced by high temperature. These findings underscore the intricate interplay between chromosomal architecture and gene expression in response to environmental stress.

## 2. Materials and Methods

### 2.1. Bacterial Culture and Heat Treatment

The bacterial strain used in this study was *Escherichia coli* MG1655, preserved in our laboratory. The LB medium was used for cultivating *E. coli* in the experiment. *E. coli* cells stored at −80 °C were inoculated in fresh LB medium at a 1:50 ratio before culturing, cultured at 37 °C overnight, and then inoculated in the LB medium at a 1:100 ratio for culturing at 37 °C. When the OD_600_ of the culture medium reached 2 (about 1.5 h), the culture medium was divided into the following two groups: the normal-temperature group and high-temperature group. The normal-temperature group was continued at 37 °C, and the culture temperature in the high-temperature group was raised to 45 °C (by placing the culture medium in a water bath of 50 °C for 1 min and 20 s) to continue. At two time points (namely, 10 min and 2.5 h after the heat treatment), 1 mL of the culture was sampled in the normal-temperature group and the high-temperature group, respectively, to prepare the 3C library, RNA-seq library, and nucleoid fluorescence imaging experiments. The reagents used in the experiments are listed in Table S1.

### 2.2. The 3C and RNA Sequencing

The method described in Lioy and co-authors’ work [9] was modified to generate the 3C library of *E. coli*. The *E. coli* cells were cultured at 45 °C for 10 min (thermal logarithmic growth phase, simplified as Therm_Log) and 2.5 h (thermal stationary growth phase, simplified as Therm_Sta) and at 37 °C for the same time points (Norm_Log and Norm_Sta) in the four groups of culture media, and 1mL of culture media in each group was sampled for the construction of the 3C and RNA sequencing libraries, respectively. The detailed process for the 3C and RNA library construction is described in Supplementary Methods, Section S2.1. The resulting 3C and RNA libraries were sequenced by Novogene company, Tianjin, China (150 bp pair-end sequencing).

### 2.3. Fluorescence Imaging of Bacterial Nucleoid

The *E. coli* culture medium was centrifuged at 3500× *g* for 10 min to remove the supernatant, and then 1 × DAPI staining solution was added to re-suspend the bacteria. After incubation at room temperature for 30 min, the supernatant was centrifuged again, washed twice with 1 × PBS, and re-suspended with 1 × PBS. Using the Nikon Ti2-E microscope for multi-channel (bright-field DIA and dark-field DAPI) imaging, the images of the two channels could be superimposed to obtain the contours of the *E. coli* cells and their nucleoids. OpenCV was used for the image recognition, and the length and width of the outer contours of the bacterial nucleoids in each fluorescence image were recorded.

### 2.4. Interaction Matrix and CID Analysis

The procedure in Lioy and co-authors’ work [9] was used to generate the chromosome interaction matrices at a 5 kb resolution (bin size) based on the data obtained from the sequencing of the 3C library. The generated interaction matrix was normalized by the SCN method [32] to facilitate the subsequent comparative analysis. The short-range interaction frequency and proportion were calculated to reflect how a target bin interacts with its neighboring bins on the genome (see Supplementary Methods, Section S2.2, for details). According to the definition in Dixon and co-authors’ work [33], the directionality index (DI) was calculated for each interaction matrix by using the script of Lioy et al. [9]. According to the definition, the DI reflects the tendency of each bin to interact with the upstream and downstream bins along the chromosomal direction. Therefore, the alternating position of the positive and negative DI value was considered as the location of the CID boundary.

### 2.5. Transcription Level and Correlation Analysis

After removing the adapter from the Illumina sequencing data, the software Cutadapt (version 3.5) was used to remove the bases of the reads with a low quality at the head and tail. Sequencing data were aligned to the reference genome (U000913.3) using the software TopHat (version 2.1.1) calling Bowtie2 (version 2.4.4). The FPKM values for each gene were calculated by combining the results of the comparison with the reference annotation information using the software Cufflinks (version 2.2.1). Since the interaction frequency data were in matrix form, with segment sizes of 5 kb as units, and the transcription levels were in gene units with large fluctuations, it was impossible to directly analyze the correlation. Firstly, we divided the transcription level data into 5 kb segments (bins) according to the location of the gene, counted the transcription level of each bin, and then calculated the log2 value of this level to make it relatively centralized and convenient for comparison. Then, the values of the diagonal in the interaction matrix were used to calculate the Z-score of the interaction frequency, and this Z-score was used for the Pearson correlation analysis with the Z-score of the transcription level. The software tools used in this study are summarized in Table S2.

### 2.6. Chromosome 3D Model Construction and Compactness Calculation

Using the EVR program [34], the normalized interaction matrices were converted to 3D structure models. The generated models could be directly visualized using PyMol. To facilitate comparison between the 3D models, we devised the following two indicators for measuring the compactness of the 3D chromosome structure: Global Compactness (GC) and Local Compactness (LC).

The GC is defined as Equation (1), as follows:

(1) $$GC=-{log}_{2}\frac{\sum_{i,j=0,i\neq j}^{n}D(i,j)}{\sum_{i,j=0,i\neq j}^{n}d(i,j)}$$

In this formula, *GC* is the Global Compactness, *i* and *j* are indices for the bins, *D* (*i*, *j*) is the Euclidean distance between bin *i* and bin *j* in the chromosome 3D structure model, and *d* (*i*, *j*) is the distance between bin *i* and bin *j* on a reconstructed circle. The details for the calculation of the *GC* are described in Supplementary Methods, Section S2.3.

The *LC* is defined as Equation (2), as follows:

(2) $${LC}_{i}=-{log}_{2}\frac{\sum_{j=i-thr}^{i+thr}D(i,j)}{\sum_{j=i-thr}^{i+thr}d(i,j)}$$

In this formula, *LC_i_* is the Local Compactness of bin *i*, *D* (*i*, *j*) is the Euclidean distance between bin *i* and bin *j* in the chromosome 3D structure model, *d* (*i*, *j*) is the distance between bin *i* and bin *j* on a reconstructed straight line within the local range of bin *i*, and *thr* is the threshold for the local range definition. The larger the *LC_i_*, the higher the chromosome compactness at the location of bin *i*. The details for the calculation of the LC are described in Supplementary Methods, Section S2.4.

## 3. Results

### 3.1. Chromosome Interaction Decreases with Linear Genomic Distance in All Conditions

**Figure 1:**
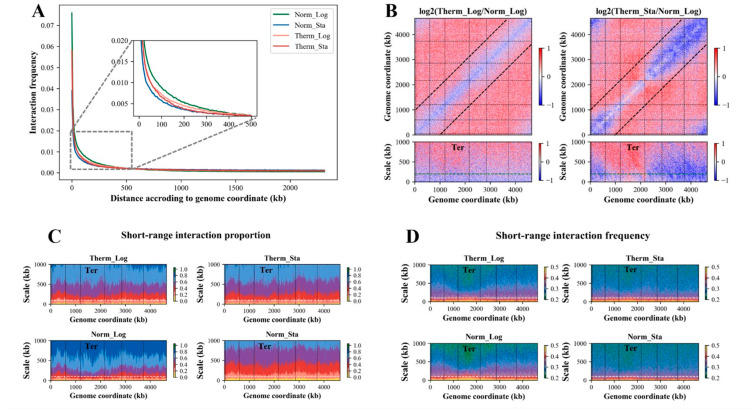
***E. coli* chromosome interactions under different growth conditions.** (**A**) Interaction frequency of the DNA segments varies with the linear genomic distance. (**B**) Heat maps for the ratio of the interaction frequency between the high- and normal-temperature growth conditions. Blue indicates a decrease in the interaction frequency under the high-temperature condition, and red indicates an increase. The green dashed lines in the figure indicate 200 kb. (**C**) Short-range (<100 kb) interaction proportions under different growth conditions. (**D**) Short-range (<100 kb) interaction frequencies under different growth conditions. The white dashed lines in the subfigures (**C**,**D**) indicate 100 kb. The black dotted vertical lines in the subfigures (**B**–**D**) indicate the boundaries of the macrodomains.

For the four groups (Norm_Log, Norm_Sta, Therm_Log, and Therm_Sta) of *E. coli* culture samples, 3C-seq experiments were conducted, yielding interaction frequencies between chromosomal DNA fragments which reflect the spatial organization of the *E. coli* chromosome under these conditions. Analysis involving the linear genomic distance and spatial interaction frequency of the DNA segments revealed notable trends. Figure 1A illustrates that the spatial interaction frequency between the DNA segments inversely correlates with the linear genomic distance under all growth conditions. Most chromosome interactions occur within a linear distance of 500 kb, and, beyond this range, the interaction frequencies decline to very low levels. Upon closer examination of the interaction frequency patterns within 500 kb, we observed that the samples from the Norm_Log group exhibited the highest interaction frequencies at equivalent linear distances, and the samples from the Norm_Sta group exhibited the lowest interaction frequencies, whereas the samples from the Therm_Log and Therm_Sta groups displayed interaction frequencies of intermediate levels.

### 3.2. The Long-Range (>200 kb) Interaction of Ter Macrodomain Increases under the Heat Stress

We calculated the ratio of the interaction matrix under heat stress and the interaction matrix under normal temperature and drew the heat maps (Figure 1B), from which the change in the *E. coli* chromosome interaction under heat stress can be obviously observed. As shown, compared with the normal-temperature condition, the interaction frequencies of the logarithmic and stationary phases under the heat-stress condition increased relatively in the range above 1000 kb, while the reduction in the interaction frequency was mainly concentrated in the range below 1000 kb, as shown by the black dashed lines in Figure 1B. The lower two subfigures in Figure 1B show the ratio of the interaction frequency over a range of 1000 kb, where the green dashed lines represent 200 kb. As shown in the left subfigure (corresponding to the Therm_Log condition), the DNA interaction frequency within 200 kb is apparently reduced. In the stationary phase under heat stress (right subfigure), the interaction frequency of the Ter macrodomain is slightly reduced in the range of 200 kb, but apparently increased beyond that, meaning that more extensive interactions occur between the Ter macrodomain and the DNA segments beyond a linear genomic distance of 200 kb.

### 3.3. Short-Range (<100 kb) Interactions Are Significantly Reduced under Heat Stress

We investigated the effect of heat stress on short-range (<100 kb) interactions within the *E. coli* genome. The short-range chromosome interactions are characterized by analyzing the frequency and proportion of the interactions between the DNA segments in proximity. The interaction frequency describes the average number of interactions between a target bin and its flanking bins (upstream and downstream) at a specific distance. The interaction proportion, on the other hand, quantifies the cumulative interaction frequency within a defined window around the target bin. These two parameters capture distinct aspects of short-range interactions.

Our analysis revealed that a significant proportion (>20%) of all interactions occur within a 100 kb window, which represents approximately 2.5% of the genome length (Figure 1C). The interaction ratio further highlights that over 60% of the target bin’s interactions originate from bins within a 500 kb window (Figure 1C). These findings suggest that chromosome interactions are primarily confined to a range of <500 kb linear genomic distance. To further explore these short-range interactions, we calculated the interaction frequency within a 1 Mb window surrounding each bin. The resulting maps (Figure 1D) clearly demonstrate a reduced interaction frequency within a range of 100 kb. Interestingly, the Ter macrodomain exhibits a distinct interaction profile. Within 100 kb, the Ter macrodomain displays a higher interaction frequency compared to other regions. However, beyond 100 kb, the interaction frequency of the Ter macrodomain drops significantly, suggesting a potential role for MatP in establishing domain-specific interaction insulation, which is in line with previous reports [35,36].

### 3.4. Transcription Level Positively Correlates with Interaction Frequency

**Figure 2:**
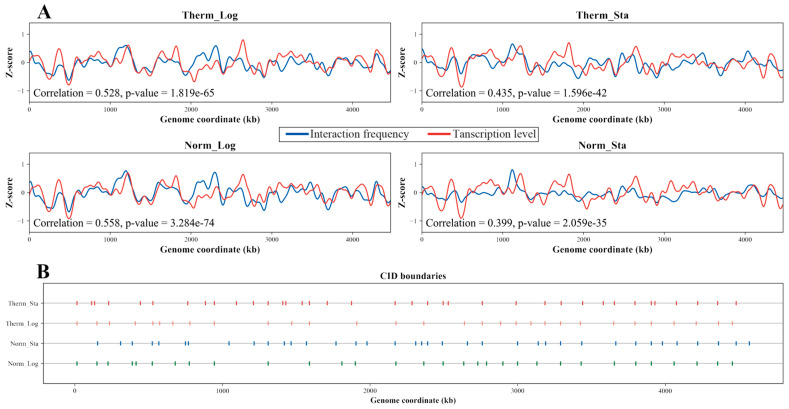
**The relationship between the DNA interaction frequency and transcription level and the CID boundaries under different growth conditions.** (**A**) Correlation between the interaction frequency and transcription level of the DNA segments under different growth conditions. Blue lines represent the Z-score of the DNA interaction frequency, and red lines represent the Z-score of the DNA transcription level. The lower left corner of each subfigure displays the correlation coefficient and corresponding significance level (*p*-value). (**B**) CID boundaries under different growth conditions. The horizontal lines represent the genomic coordinates, and the points on each line represent the CID boundaries.

We explored the relationship between chromosome interaction and gene expression in *E. coli* under different growth conditions. To analyze the correlation between the DNA interaction frequency and transcription level, we assigned the transcription levels of the genes into 5 kb bins (see Section 2 for details). Then, the average transcription level of each bin and the Z-score of the interaction frequency in the upstream and downstream 10 kb range of the bin were calculated. The Z-score curves of the interaction frequency and the corresponding transcription levels are shown in Figure 2A for the four growth conditions, respectively. As can be seen, there are clear positive correlations between the trends of the interaction frequency and the transcription level for the samples from these conditions. The correlation coefficients for the two log-phase samples are greater than 0.5, and the correlation coefficients for the two stationary-phase samples are around 0.4, and all of them are highly significant (*p*-value near zero for each sample). Changes in the growth conditions affect the interaction frequency and the transcription level of the DNA, while the correlation between them remains positive under all conditions.

### 3.5. Differential CID Boundary Genes Are Related with Cell Wall and Membrane

We identified the Chromosome Interaction Domains (CIDs) in the *E. coli* genome by calculating directionality index (DI) [33]. For the four growth conditions, the distribution of the CID boundaries across the genome for each condition is depicted in Figure 2B. Interestingly, while the total number of CIDs remains relatively constant between the normal- and high-temperature logarithmic phases (31 vs. 30), their boundary locations differ. Gene Ontology (GO) enrichment analysis of the genes located at these differential boundaries revealed a significant enrichment for the terms related to lipopolysaccharides (LPSs) and oligosaccharides (Figure S3). As LPSs are a crucial component of the *E. coli* cell wall, this result may suggest that heat stress alters the LPS structure, triggering a signaling cascade that activates various cellular stress responses. The analysis of the stationary phases identified 37 CIDs in both temperature conditions. Notably, the stationary phases displayed a higher density of CID boundaries around the Ter macrodomain compared to the logarithmic phases, accompanied by a general decrease in the CID size. Furthermore, GO enrichment analysis of the genes located at the differential CID boundaries in the high-temperature stationary phase identified an enrichment for the terms associated with the transmembrane transport (Figure S3). This observation is consistent with the notion that heat stress can damage the cell wall, necessitating enhanced transmembrane transport activity for intracellular repair processes. These findings collectively support a strong link between changes in growth conditions and the dynamic reorganization of the *E. coli* chromosome structure.

### 3.6. The Global Compactness of E. coli Chromosome Is Higher in Stationary Phase and High-Temperature Environment

**Figure 3:**
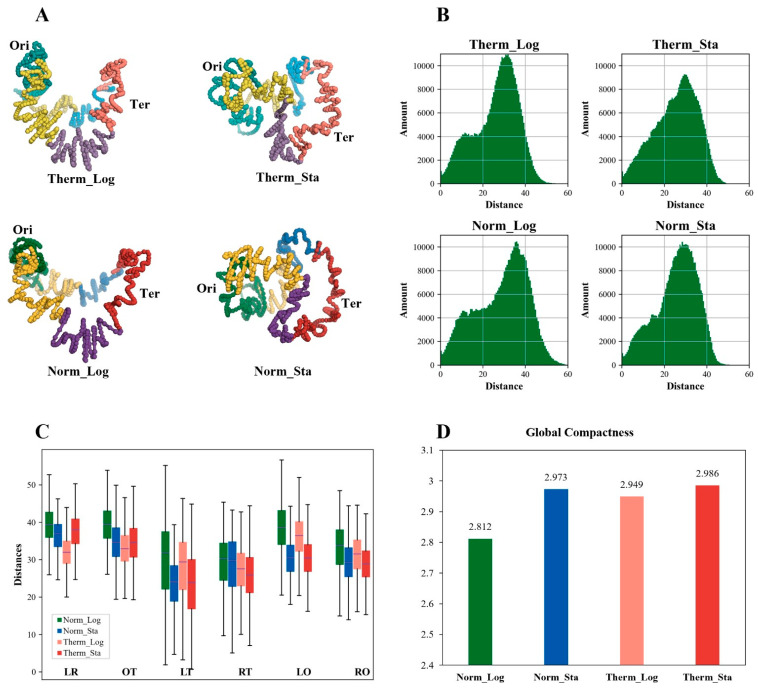
**The 3D structural features of the *E. coli* chromosome under different growth conditions.** (**A**) The 3D structural models of the *E. coli* chromosome under different growth conditions. In the Norm_Log and Norm_Sta models, green is the Ori macrodomain, red is the Ter macrodomain, purple and blue are the Left and Right macrodomains, respectively, and yellow is the non-structured regions; in the Therm_Log and Therm_Sta models, lighter colors are used correspondingly. (**B**) Distribution of the distance between points (bins) in these 3D models. (**C**) Spatial distances between the bins of the macrodomains in the 3D models of the *E. coli* chromosome under different growth conditions. O: Ori macrodomain; T: Ter macrodomain; L: Left macrodomain; R: Right macrodomain. OT represents the distances between the bins in the Ori macrodomain and the bins in the Ter macrodomain, and so on. (**D**) Global Compactness of the *E. coli* chromosome models under different growth conditions.

To further explore chromosome changes, we employed the EVR program [34] to generate 3D structure models of the *E. coli* chromosome under various growth conditions (Figure 3A). The models depict four macrodomains (Ori, Ter, Left, and Right) and two non-structured regions. All models share a crescent-shaped ring structure with numerous folds. Notably, Ori and Ter occupy opposite ends of the models, with the Left and Right macrodomains flanking Ter but separated without significant intertwining.

While all of the models exhibit a crescent-shaped ring, subtle differences exist upon closer inspection. The log-phase models appear highly similar, although the Therm_Log model displays a slightly closer proximity between Ori and Ter. The stationary-phase models appear smaller than their log-phase counterparts, with a significantly reduced distance between Ori and Ter. To quantify these observations, we analyzed the distance distribution for each point (bin) pair within the 3D models (Figure 3B). The log-phase models exhibit a near-bimodal distribution, with a dominant peak around 30–40 units and a less prominent peak near 10 units. In contrast, the stationary-phase models display a single peak at 30–40 units. This suggests a decrease in the frequency of the short distance (around 10 units) and an increase in the frequency of the intermediate distance (around 20 units) in the stationary-phase models, particularly under heat stress. To further substantiate these findings, we analyzed distance variations between macrodomain bins (namely, the bins belonging to each macrodomain, respectively) within the models (Figure 3C). The data reveal the following consistent trend: distances between any two macrodomains tend to be smaller in the high-temperature log-phase models compared to the normal-temperature log-phase models. Additionally, the distances between the bins within each macrodomain also show a decreasing trend for these macrodomains, suggesting a genome-wide trend of chromosome condensation under heat stress.

To quantify the overall chromosome compactness, we defined a metric termed Global Compactness (GC). The GC calculation involves reconstructing the chromosome model into a circle and then calculating the negative logarithm of the ratio between the sum of the distances in the original model and the sum of the distances in the reconstructed circle (see Methods and Supplementary Methods for details). Higher GC values indicate greater compactness or condensation. The GC values for the Norm_Log and Norm_Sta models were 2.812 and 2.973, respectively, and 2.949 and 2.986 for the Therm_Log and Therm_Sta models, respectively (Figure 3D). These results support the observation that chromosomes under high temperature are more compact than those under normal temperature at the same growth stage. Additionally, the degree of chromosome condensation is higher in the stationary phase compared to the logarithmic phase under the same temperature condition. This aligns with previous studies employing sucrose density gradient centrifugation to isolate *E. coli* nucleoids at different growth stages, which reported a more compact nucleoid structure in the stationary phase [37]. In conclusion, both the transition to the stationary phase and exposure to heat stress promote a higher degree of overall chromosome condensation in *E. coli*.

### 3.7. No Simple Relationship between Chromosome Global Compactness and the Nucleoid Size of E. coli Cell

**Figure 4:**
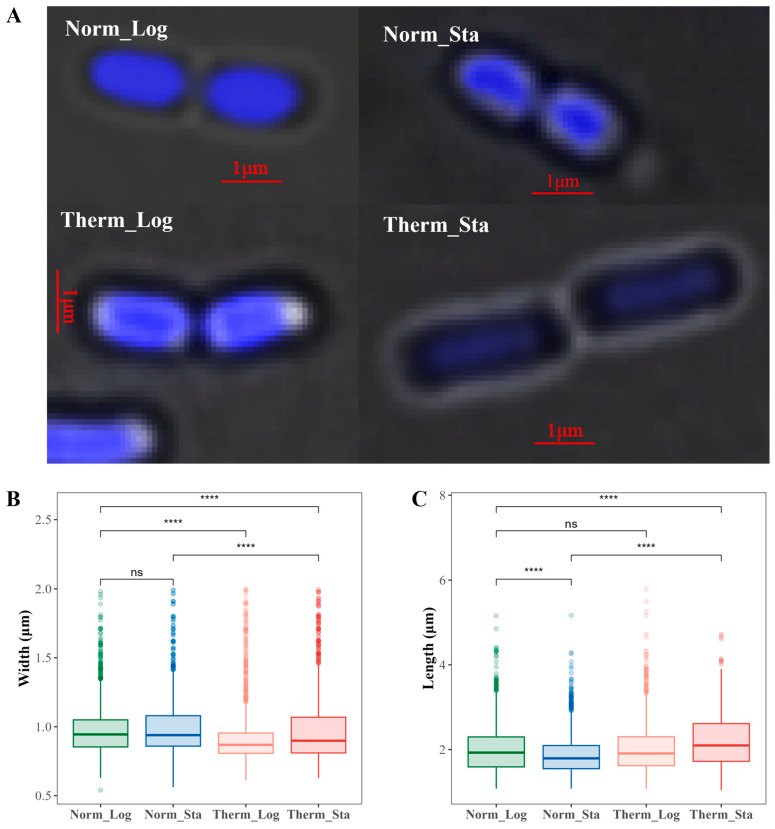
**Comparison of the *E. coli* nucleoid morphology in different growth conditions.** (**A**) Typical microscopic images of the *E. coli* nucleoid under various growth conditions. The nucleoids of *E. coli* were stained blue by DAPI. (**B**) Statistical results of the *E. coli* nucleoid width under different growth conditions. (**C**) Statistical results of the *E. coli* nucleoid length under different growth conditions. In B and C, significance levels are denoted by the following symbols: ns for *p* > 0.05 and **** for *p* ≤ 0.0001.

*E. coli* typically appears as a short, straight rod-shaped bacterium. However, high-temperature stress can influence the cell size. Previous studies [38,39] have reported an increase in the *E. coli* cell length at elevated temperatures (up to 50 °C). Consistent with these reports, DAPI staining and fluorescence microscopy revealed that *E. coli* in the high-temperature environment displayed a changed morphology (Figure 4A). In order to accurately compare the size variations in the *E. coli* nucleoid under different growth conditions, we utilized microscopic image analysis to measure the width and length of each nucleoid. Approximately 2000 *E. coli* cells were analyzed for each growth condition. The statistical result in Figure 4B indicates that the widths of the *E. coli* nucleoid do not exhibit significant change between the logarithmic and stationary phases at normal temperature. Conversely, in a high-temperature environment, the widths of the *E. coli* nucleoid decrease significantly for both the logarithmic and stationary phases, with significantly higher values in the stationary phase than in the logarithmic phase. The statistical results for the length are presented in Figure 4C, revealing that, compared to the logarithmic phase, the lengths of the *E. coli* nucleoid are notably shorter during the stationary phase at normal temperature. However, there is no significant difference in the length for the logarithmic phase between high and normal temperatures; meanwhile, the nucleoid lengths are significantly larger in the high-temperature stationary phase compared to the other three phases. The 3D genomic data demonstrate that *E. coli* chromosomes exhibit higher GCs in the high-temperature environment, which may be associated with the reduced width of the *E. coli* nucleoid in such conditions. Furthermore, our results also indicate that the GC of the *E. coli* chromosome in the stationary phase was greater than that in the logarithmic phase for both normal- and high-temperature conditions (more pronounced in the former condition); meanwhile, the length of the nucleoid in the stationary phase was smaller under normal temperature and larger under high temperature compared to that in the logarithmic phase. Considering all of these results together, there is no simple relationship between the chromosome GC and the nucleoid size in *E. coli* cells, meaning that the GC is not a simple reflection of the nucleoid size but an indicator of the intrinsic properties of the chromosome.

### 3.8. The Local Compactness of E. coli Chromosome Is Affected by Growth Condition

**Figure 5:**
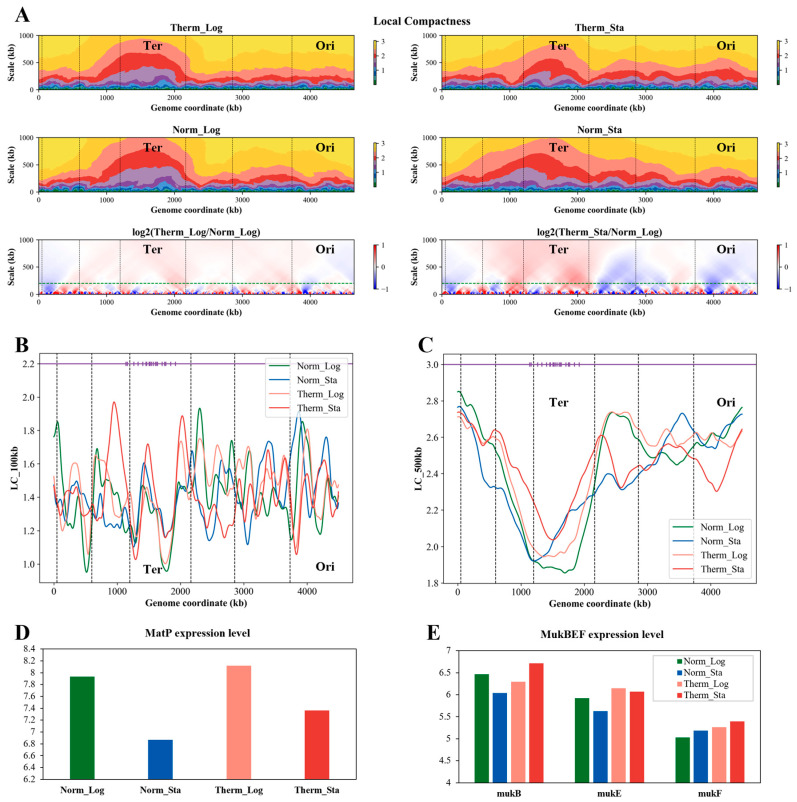
**Local Compactness of the *E. coli* chromosome and related features.** (**A**) The Local Compactness of the *E. coli* chromosome 3D structures under different growth conditions and its ratio between high and normal temperatures. The green dashed lines in the bottom two subfigures of figure A indicate the scale of 200 kb. (**B**) The Local Compactness in the 100 kb range of the *E. coli* chromosome 3D structures under different growth conditions. (**C**) The Local Compactness in the 500 kb range of the *E. coli* chromosome 3D structures under different growth conditions. In subfigures (**B**,**C**), the lines of different colors correspond to different growth conditions. The dots on the purple horizontal lines represent the location of MatS, which is the binding site of MatP. The black dashed vertical lines in the subfigures (**A**–**C**) represent the boundaries of the macrodomains. (**D**,**E**) Transcription levels of MatP and MukBEF in *E. coli* under different growth conditions, respectively.

To further characterize the change in the chromosome 3D structure, we defined a metric called Local Compactness (LC). The LC is calculated at various scales (up to 1000 kb) across the entire genome. Figure 5A depicts the heatmap representing the genome-wide LC for all analyzed scales. As the calculation scale increases, the overall LC increases, with a larger fluctuation observed at smaller scales. Notably, the LC exhibits an uneven distribution throughout the genome at any scale, with the Ter macrodomain displaying significantly lower compactness compared to other regions. This pattern persists across all growth conditions (heat stress or stationary phase). To evaluate the influence of the growth condition on the LC, we calculated the logarithmic ratio of the LC between heat stress and normal temperature (Figure 5A, lower panels). At smaller scales (less than 50 kb), the LC fluctuates considerably. However, at larger scales (more than 200 kb), the LC of the Ter macrodomain and its flanking regions significantly increases under high temperature, while the LC of the Ori macrodomain and its flanks decreases, and this decrease is more pronounced in the stationary phase.

We further analyzed the LC at two specific scales (100 kb and 500 kb) across all of the four growth conditions, with the MatS location highlighted in purple (Figure 5B,C). The 100 kb LC profiles display significant fluctuations, with less prominent differences observed at the Ter macrodomain boundaries. However, the 500 kb profiles reveal a substantial LC increase at the Ter macrodomain boundaries under heat stress. This suggests a potential for increased interaction between the Ter macrodomain and distant DNA segments under high-temperature conditions, which is consistent with the long-range interaction frequency analysis above. MatP, a key NAP, plays a crucial role in Ter macrodomain organization by specifically recognizing and binding to MatS sites on DNA. As shown in Figure 5D, MatP expression is upregulated under high-temperature stress. Conversely, the MukBEF complex, involved in long-distance chromosome organization [11], exhibits varying degrees of upregulation under heat stress (Figure 5E; see also Table S3 for a statistic). We hypothesize that high-temperature stress alters MatP expression, leading to its enhanced binding to MatS sites and the increased compactness of the Ter macrodomain. The MukBEF complex, on the other hand, might be more involved in mediating long-distance chromosome interactions under heat stress, potentially contributing to a reduction in the distances between the Ter macrodomain and other macrodomains (Figure 3C).

### 3.9. The Local Compactness Is Negatively Correlated with Transcription Level

**Figure 6:**
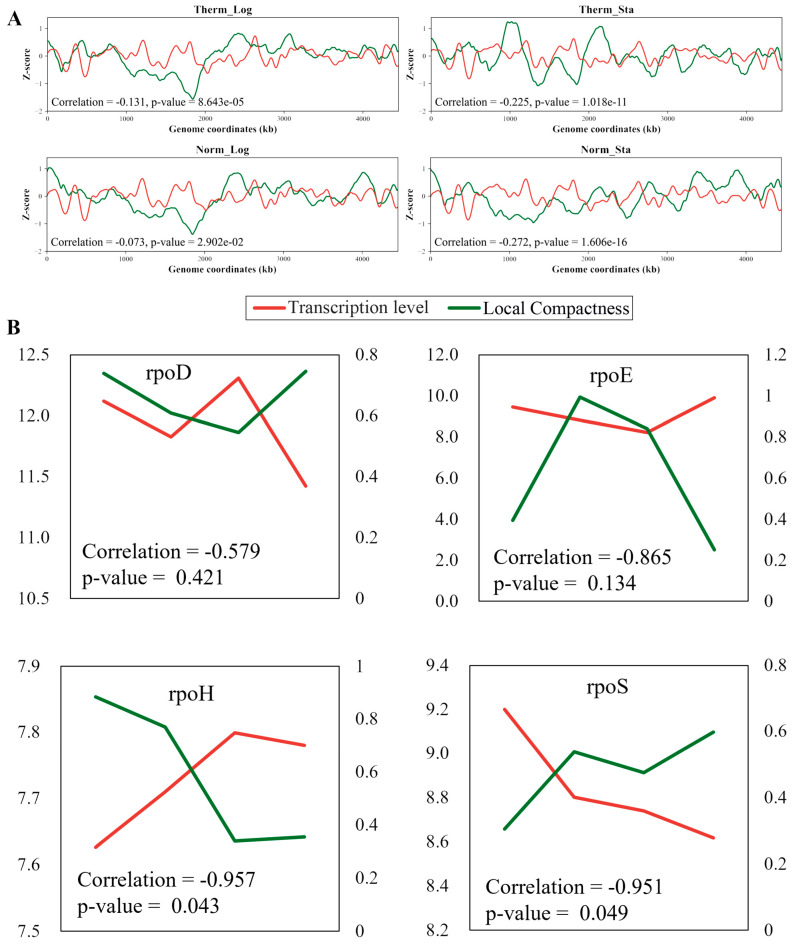
**Correlation between the Local Compactness and transcription level.** (**A**) The genome-wide correlation between the Local Compactness and transcription level under different growth conditions. (**B**) The correlation between the transcription levels of four sigma factors and their Local Compactness (where the sigma factor genes reside in the linear genome). Green lines correspond to the Local Compactness; red lines correspond to the transcription level.

The Z-score curves illustrating the relationship between the LC and transcription level are depicted in Figure 6A. We observed a negative correlation between the LC and transcription level across the entire genome, with correlation coefficients being small but significant (*p* < 0.05). Heat stress triggers the accumulation of misfolded proteins in *E. coli*, and the refolding or degradation of these aberrant proteins necessitates the involvement of numerous heat shock proteins (HSPs) [26]. Sigma factors, namely, σ^70^, σ^32^, σ^S^, and σ^E^, play a critical role in the transcriptional regulation of HSP genes, where σ^70^ is encoded by rpoD, σ^32^ is encoded by rpoH, σ^S^ is encoded by rpoS, and σ^E^ is encoded by rpoE. We analyzed the LC changes in the chromosome regions harboring the genes (rpoD, rpoH, rpoE, and rpoS) encoding these sigma factors within the 3D models of *E. coli* chromosomes under various growth conditions (Figure 6B). These analyses revealed a strong negative correlation between the LC and the corresponding transcription levels of the sigma factor genes. Overall, the correlation between the LC and transcription level is negative and weak, but sometimes strong for specific genes.

## 4. Discussion

Bacteria constantly encounter environmental fluctuations, including temperature and others. These changes trigger stress responses, impacting their internal physiology and biochemistry, including transcriptional expression. These cellular alterations can directly or indirectly influence the chromosome structure. The 3C technology and its derivatives provide valuable tools to resolve the spatial relationships between DNA segments on chromosomes. This study successfully employed 3C-seq to capture the chromosome interaction data and built 3D structure models of *E. coli* chromosomes. Furthermore, two metrics of the GC and LC were defined for the 3D structural model to elucidate the changes in chromosome conformation. It was observed that, under high-temperature stress, the short-range interaction frequency of the chromosome decreased while the GC of the chromosome increased. The long-range interaction frequency of the Ter macrodomain increased, while its LC decreased. Correlations were found between the transcription level and the short-range interaction frequency. These findings shed light on the impact of heat stress on the structure and function of *E. coli* chromosomes.

NAPs play a crucial role in the *E. coli* chromosome architecture and gene expression regulation. Environmental stress can affect the expression of NAPs. Figure S4 depicts the transcription levels of key NAPs (HU, Fis, CbpA, H-NS, and StpA) under various growth conditions. CbpA, a non-sequence-specific DNA-bending protein [40], is negatively regulated by Fis during rapid growth [41]. Fis is known to mediate interactions between DNA segments exceeding 100 kb, HU is thought to facilitate interactions in the range of 50–280 kb, and H-NS is involved in long-range interactions (>250 kb) [9]. StpA is a kind of H-NS-like NAP that can form rigid DNA filaments with the function of a transcription suppressor, and its non-specific binding affinity is five times higher than H-NS [42,43,44,45]. Thus, the observed decrease in the short-range interaction frequency under heat stress may be attributed to the upregulation of Fis and StpA transcription levels, and the decrease in the CbpA and HU transcription levels.

It is well known that the growth rate of *E. coli* is slower in the stationary phase than in the logarithmic phase, and the growth rate also slows down under high-temperature stress conditions [46]. In our results, it was found that the GC of the *E. coli* chromosome increased under stationary and high-temperature conditions. Therefore, the chromosome GC and the growth rate of *E. coli* may be related with each other. However, combining the 3D genomic and nucleoid imaging results, we found that there is no simple correspondence between the chromosome GC and the nucleoid size. Therefore, more work is needed to clarify the relationship between the growth rate of *E. coli* and the conformational change in its chromosome. Moreover, conventional studies on the heat shock response revealed that many sigma factors are directly involved in the regulation of the temperature response [47], while sigma factors are also responsible for the regulation of the gene transcription level [48]. Indeed, in our results, there is a close link between the LC and transcription level of sigma factors, with the former (LC) apparently affected by the growth condition, providing a new clue for understanding the role of sigma factors in the regulation of the temperature response.

In conclusion, the joint analysis of 3D genomics and transcriptomics revealed the impact of heat stress on the structure and function of *E. coli* chromosomes, which is crucial for understanding the molecular mechanism of the 3D genome structure and heat adaptation in *E. coli*. The GC and LC metrics proposed in this work provide valuable tools for measuring and comparing chromosome 3D structures in future studies.

## Data Availability

The 3C-seq and RNA-seq FASTQ files were deposited in the Gene Expression Omnibus (accession no. GSE211825). The scripts used for analyzing the 3C data have been deposited to Github (https://github.com/mbglab/Eco3Dheat).

## Supplementary Materials

The following supporting information can be downloaded at: https://www.mdpi.com/article/10.3390/microorganisms12061229/s1, Supplementary Information, including supplementary materials, methods, and results. Figure S1: Global Compactness calculation diagram; Figure S2: Global Compactness and Local Compactness of six theoretical models; Figure S3: GO enrichment results of chromosome differential CID boundary genes in *E. coli* under heat stress; Figure S4: Transcription levels of some *E. coli* NAPs under different growth conditions; Table S1: Regents; Table S2: Software; Table S3: The expression levels of some NAPs involved in Ter macrodomain organization.
